# Morphological and genetic evidence supports the separation of two *Tapinoma* ants (Formicidae, Dolichoderinae) from the Atlantic Forest biome

**DOI:** 10.3897/zookeys.1033.59880

**Published:** 2021-04-22

**Authors:** Mayron E. Escárraga, John E. Lattke, Marcio R. Pie, Roberto J. Guerrero

**Affiliations:** 1 Facultad de Ciencias Básicas, Programa de Biología, Universidad del Magdalena, Carrera 32 # 22-08, Santa Marta, Magdalena, Colombia Universidad del Magdalena Santa Marta Colombia; 2 Departamento de Zoologia, Universidade Federal do Paraná, Curitiba, Brazil Universidade Federal do Paraná Curitiba Brazil

**Keywords:** Cryptic diversity, haplotype network, intraspecific variation, mitochondrial DNA, neglected taxon, phylogenetic reconstruction

## Abstract

The taxonomic boundaries of many Neotropical ant species of the genus *Tapinoma* are still unclear. *Tapinoma atriceps* and *T. atriceps breviscapum* are two morphologically similar taxa which occur sympatrically in the southern Atlantic Forest of Brazil. Some characters such as the scape length and head shape suggest that these taxa may be different species. We used DNA analysis and morphological evidence, including scanning electron microscopy, to evaluate the taxonomic validity of these taxa. We found distinct morphological characteristics that allow separating them as two different species, *Tapinoma atriceps* and *Tapinoma breviscapum***status novo**, and this decision is supported by the DNA results, where *Tapinoma atriceps* was recovered as a lineage independent of *T. breviscapum*.

## Introduction

*Tapinoma* Foerster is an ant genus distributed worldwide, with 69 known species (Bolton 2021) mostly in the tropics. Of these 69 valid taxa, 19 species, including several undescribed ones, are recognized in the Neotropical region (R.J. Guerrero unpublished data). The workers are morphologically recognized by their small size and reduced petiolar node covered by the first gastral tergite (Shattuck 1992), although these characteristics are convergent with *Technomyrmex*. *Tapinoma* workers, however, can be differentiated from those of *Technomyrmex* in that the latter present five gastral tergites, while in *Tapinoma* only four tergites are present. *Tapinoma atriceps* Emery, 1888, which was was described as a pale-yellow ant with a brown head and gaster from specimens collected at an unknown locality in the state of Rio Grande do Sul (Brazil), is considered vaguely similar to *Tapinoma melanocephalum* (Fabricius, 1793). *Tapinoma atriceps breviscapum* Forel, 1908 was later described from specimens with a similar color pattern to *T. atriceps* and collected in the state of São Paulo, but it is recognizable by having a longer and more rectangular head and shorter scapes.

According to published records for Brazil, *T. atriceps* occurs in the states of Mato Grosso do Sul (Demetrio et al. 2017), Rio de Janeiro (Eidmann 1936; Santos et al. 2019), São Paulo (Forel 1908), Paraná (Lozovei 2001), Santa Catarina (Farneda et al. 2007; Lutinski et al. 2008, 2013; Schmid et al. 2010; Gadelha et al. 2016), and Rio Grande do Sul (Emery 1888). Outside of Brazil, *T. atriceps* has been reported from rainforest in Misiones, Argentina (Hanisch et al. 2015) and Canindeyú, Paraguay (Wild 2007a). These records show that it mainly inhabits vegetation of the Atlantic Forest in southeastern South America. In contrast with the many records of *T. atriceps*, there are no published records for *T. a. breviscapum* after its description, perhaps because of misidentifications with *T. atriceps*.

There is no recent taxonomic revision of *Tapinoma* nor a phylogenetic framework to understand the relationships among the Neotropical species of *Tapinoma*, nor DNA sequences for many species. Given this situation, the identity and boundaries of species like *T. atriceps*, as well as the validity of the subspecies *T. a. breviscapum*, are unclear. The integration of molecular data along with the examination of morphology could provide a clear resolution of taxonomic limits in these taxa. Here, we used morphological and DNA evidence to evaluate the taxonomic validity of *T. atriceps* and *T. atriceps breviscapum*.

## Material and methods

We examined 180 specimens of *Tapinoma atriceps* and *T. a. breviscapum*, including workers, queens, and males. A syntype worker of *Tapinoma atriceps* was examined from high-resolution photographs available at http://www.antweb.org (specimen code CASENT0904029). Three syntype workers of *T. a. breviscapum* from the Museum d’Histoire Naturelle (**MHNG**) were also examined.

### Institutional acronyms

The collection abbreviations are taken from Evenhuis (2020) except for those ones marked with an asterisk (*). The material upon which this study is based is located and/or was examined at the following collections or institutions:

content-type="institution" **ALWC** Alexander L. Wild personal collection, Austin, Texas, USA;

**CPDC**Centro de Pesquisas do Cacau, Comissão do Plano de Lavoura, Itabuna, Bahia, Brazil;

**DZUP**Coleção Entomológica Padre Jesus Santiago Moure, Universidade Federal do Paraná, Curitiba, Paraná, Brazil;

**MHNG**Muséum d’Histoire Naturelle, Geneva, Switzerland;

**MSNG**Museo Civico di Storia Naturale “Giacomo Doria”, Genoa, Italy;

**MZSP**Museu de Zoologia, Universidade de São Paulo, São Paulo, Brazil;

**PSWC**Philip S. Ward Collection, University of California, Davis, California, USA*;

**USNM**National Museum of Natural History, Smithsonian Institution, Washington, DC, USA;

**WEMC**William and Emma Mackay Collection, University of Texas, El Paso, Texas, USA*.

### Sampling and geographic origin

To obtain fresh samples for DNA and morphological analyses, we collected specimens in five Brazilian localities in the states of Minas Gerais, Paraná, and Santa Catarina between April 2016 and June 2017. Additional ethanol-stored specimens from Rio Grande do Sul and Misiones (Argentina) were included (Table 1). Field collections were carried out by searching actively in the vegetation, opening hanging dry twigs and standing branches. These specimens are deposited in the DZUP. Fieldwork was approved by the Instituto Chico Mendes de Conservação da Biodiversidade (ICMBio), Sistema de Autorização e Informação em Biodiversidade (SISBIO) (approval number 25948-3).

### Measurement and indices definitions

Morphological descriptions and measurements of specimens were performed using a Nikon SMZ 740 binocular stereomicroscope equipped with a micrometer at magnifications of 96×. Morphometric characters were examined in workers, queens, and males. The following measurements and indices were used (all measurements expressed in millimeters):

Head length (HL): in full-face view, the length between the mid-point of the anterior margin of the clypeus to the mid-point of a line tangent to the posterior margin of the head.

Head width (HW): in full-face view, the maximum width between the lateral margins of the head including the eyes which are within the cephalic capsule. In males, HW is recorded above compound eyes.

Scape length (SL): the maximum length of the scape excluding the basal constriction.

Weber'S, length (WL): in lateral view of the mesosoma, greatest distance from the approximate inflection point, where the pronotum curves into the cervical shield, to the posterior basal angle of the metapleuron.

Cephalic index (CI): HW/HL×100.

Scape index (SI): SL/HL×100.

The syntype worker of *Tapinoma atriceps* was measured from high resolution photographs using the program ImageJ v. 1.3 (Schneider et al. 2012). In the results, the measurements are presented as the mean value, followed by the standard deviation, with the minimum and maximum values between parentheses. Morphological terminology for wings follows Yoshimura and Fisher (2011).

### Photographic resources and distribution map

High-resolution photographs of the specimens were captured using a Leica MZ16 stereomicroscope with a Leica DFC 500 camera, and final images were generated with Leica LAS 3D viewer LAS Montage v. 4.7. Integument surface and pilosity were examined using scanning electron microscopy (SEM) images generated with a JEOL JSM 6360-LV microscope under low vacuum (12–18 Pa) and a voltage acceleration of 15kV. Figure plates were designed with InkScape v. 0.92 (available at http://www.inkscape.org).

The distribution map of the species was made with Quantum GIS v. 3.8 (QGIS Development Team 2017) using locality records of the examined material. The coordinate system used was UTM WGS84. When available, geographic coordinates were taken from the labels, otherwise, the coordinates were estimated using Google Maps by choosing a central point from the cited locality. For the final map composition, we used a polygon of the Atlantic Forest from the World Wild Fund (Olson et al. 2001). Biology information was extracted from literature, field observations, and label data.

### Designation of type specimens

Lectotypes of *Tapinoma atriceps* and *Tapinoma breviscapum* were designated by taking a worker from the syntype series of each of these taxa. By affixing a single specimen as the name-bearing type of *T. atriceps* and a single specimen as the name-bearing type of *T. breviscapum* (Art. 74, ICZN 1999), it “permanently deprives all other specimens that were formerly syntypes of that nominal taxon of the status of syntype; those specimens then become paralectotypes” (Art. 74.1.3, ICZN 1999).

### Statistical analysis

For evaluating possible relationships between morphometric characters in the workers of both taxa, especially those associated with the head, we constructed bivariate graphs (e.g., HL vs SL). Considering that the length and width of the head or the length of the scape appear to show variability between the workers and queens of *T. atriceps* and *T. atriceps breviscapum*, we analyzed the variability of HL, HW, SL, and WL between these two taxa using a parametric or a non-parametric comparison test, depending on the results of the Normality test of the data. For the latter, each of these morphometric characters were analyzed with a Shapiro-Wilks test. For the worker data set (*n* = 44), only SL showed normality (*W* = 0.94, *p* = 0.0875, α = 0.05; Suppl. material 1: Table S1), while for queens only SL and HW showed normality (Suppl. material 1: Table S1), although this last result may be biased by the small number of samples (*n* = 10). None of the measurements in the males showed normality. The variability of these morphometric traits (i.e., non-overlapping differences) in workers and queens were analyzed using the Student's, t-test (*T*) with different sample sizes and different variances at a significance level of α = 0.05. In measurements with no normality, the difference of the two samples was evaluated with a Wilcoxon signed rank test at a significance level of α = 0.05. In the latter case, statistically significant differences were never found for any of the castes. All statistical analyses were performed in InfoStat v. 2020 (Di Rienzo et al. 2020).

### DNA extraction, amplification, and sequencing

DNA was extracted, amplified, and sequenced from eight workers of *T. atriceps* from seven localities and one worker of *T. a. breviscapum* from one locality in the Serra do Cipó, Minas Gerais, which is the only colony we managed to collect. Unfortunately, all other studied samples of *T. a. breviscapum* were unsuitable for DNA extraction. DNA was extracted from entire specimens using a GenElute TM Blood Genomic Extraction Kit (Sigma-Aldrich, Darmstadt, Germany) following the kit instructions. From each sample one worker was conserved as a voucher (Table 1). Standard polymerase chain reaction (PCR) methods were used to amplify partial fragments of the mitochondrial gene Cytochrome c oxidase subunit I (COI), the nuclear genes Long-wavelength Rhodopsin (LW Rh) and *wingless* (Wg), and an exon-primed intron-crossing marker (EPIC). Primers can be found on Table 2.

DNA amplification was performed to a final volume of 25 µL. The PCR conditions for the COI marker were: 94 °C for 2 min, followed by 32 cycles of 94 °C for 45 s, 45 °C for 45 s, and 72 °C for 1 min, then 72 °C for 5 min. PCR conditions for Wg: 95 °C for 5 min, followed by 35 cycles of 92 °C for 1 min, 58 °C for 1 min, and 70 °C for 2 min, then 72 °C for 6 min. PCR conditions for the LW Rh marker: 95 °C for 5 min, followed by 35 cycles of 94 °C for 1 min, 56 °C for 1 min, and 70 °C for 1 min, then 72 °C for 5 min. PCR conditions for EPIC: 95 °C for 5 min, followed by 35 cycles of 92 °C for 1 min, 60 °C for 1 min, 70 °C for 1 min, then 72 °C for 6 min. All the sequences generated in this study were deposited in GenBank and the accession numbers are listed in Table 1.

**Table 1.** List of specimens used in phylogenetic reconstruction and haplotype network from molecular data. Geographic information for each of the samples is recorded. The GenBank codes of these specimens are also included.

Taxon	Country	State/province	Locality	Latitude/Longitude	Collection date	Voucher code	GenBank accession number				Haplotype code
							COI	LW Rh	WG	EPIC 1281	
*Tapinoma atriceps*	Argentina	Misiones	Parque Teyú Cuaré	27°10.248'S, 55°21.720'W	28 Dec. 2007	DZUP 548801	MG920282	MN294972	-	-	H07
*Tapinoma breviscapum*	Brazil	Minas Gerais	Serra do Cipó	19°14.874'S, 43°33.054'W	25 Jun. 2017	DZUP 548800	MG920285	MN294973	MN294963	MT375619	H08
*Tapinoma atriceps*	Brazil	Minas Gerais	Serra do Cipó	19°15.264'S, 43°31.002'W	26 Jun. 2017	DZUP 548799	MG920286	MN294971	MN294964	MT375620	H04
*Tapinoma atriceps*	Brazil	Paraná	Antonina	25°18.354'S, 48°39.678'W	10-13 Jul. 2016	DZUP 548798	MG920278	MN294965	MN294957	MT375614	H01
*Tapinoma atriceps*	Brazil	Paraná	Antonina	25°17.796'S, 48°39.594'W	29 Oct. 2016	DZUP 548797	MG920280	MN294967	MN294959	-	H05
*Tapinoma atriceps*	Brazil	Paraná	Guaraqueçaba	25°09.816'S, 48°17.880'W	08 Oct. 2016	DZUP 548784	MG920281	MN294968	MN294960	MT375616	H06
*Tapinoma atriceps*	Brazil	Paraná	Paranaguá	25°35.016'S, 48°32.496'W	28 Apr. 2016	DZUP 548786	MG920279	MN294966	MN294958	MT375615	H02
*Tapinoma atriceps*	Brazil	Rio Grande do Sul	Porto Alegre	30°10.824'S, 51°06.078'W	27 Dec. 2016	DZUP 548788	MG920283	MN294969	MN294961	MT375617	H03
*Tapinoma atriceps*	Brazil	Santa Catarina	Florianópolis	27°35.928'S, 48°25.962'W	25 Feb. 2017	DZUP 548789	MG920284	MN294970	MN294962	MT375618	H01

**Table 2.** DNA primer sequences and references used for PCR amplification.

Genetic marker	Primer name	Sequence (5'–3')	Reference
COI	LCO1490	GGTCAACAAATCATAAAGATATTGG	Folmer et al. (1994)
COI	HCO2198	TAAACTTCAGGGTGACCAAAAAATCA	Folmer et al. (1994)
Wg	Wg578F	TGCACNGTGAARACYTGCTGGATGCG	Ward and Downie (2005)
Wg	Wg1032R	ACYTCGCAGCACCARTGGAA	Abouheif and Wray (2002)
LW Rh	LR143F	GACAAAGTKCCACCRGARATGCT	Ward and Downie (2005)
LW Rh	LR639ER	YTTACCGRTTCCATCCRAACA	Ward and Downie (2005)
EPIC 1281	ant.1281F	GACGCAGGTTGYAACGAAATCAC	Ströher et al. (2013)
EPIC 1281	ant.1281R	GCCRCTAATATCCAGCTTCACGAG	Ströher et al. (2013)

### Analysis of genetic data

Consensus sequences were obtained with Staden Package (Staden 1996). The intronic region of LW Rh (LW Rhi) was separated and treated as a different marker than the exonic sequences (LW Rhe). For each marker, the sequences were aligned with Muscle (Edgar 2004) and then calculated nucleotide composition and *p*-distance in Mega X (Kumar et al. 2018). Composition for COI was analyzed by constructing a haplotype network using TCS network (Clement et al. 2002) in PopART v. 1.7 software (Leigh and Bryant 2015).

DNA sequences for the species *Tapinoma opacum* Wheeler & Mann, 1914 and *T. melanocephalum* (Fabricius, 1793) were downloaded from Genbank and used as outgroups (Suppl. material 2: Table S2). Of these taxa, *T. melanocephalum* was selected to root the phylogenetic tree as one phylogenetic analysis previous suggest that *T. opacum* and *T. atriceps* are nesting in a clade of Neotropical species which is sister to the Nearctic clade (*T. sessile* + *T. schreiberi*), while *T. melanocephalum* is phylogenetically distant from those clades (R.J. Guerrero unpublished data). For the phylogenetic analysis, each of the four aligned loci were analyzed separately in a Bayesian phylogenetic framework using MrBayes v. 3.2.6. Each of the three genes was divided by codon position (position 1 + 2 and 3), along with the LW Rh intron, which was treated as another partition for this gene. The partitions and the best substitution models used by MrBayes (Suppl. material 3: Table S3) were determined using Akaike information criterion (AIC) with PartitionFinder v. 2.1 (Lanfear et al. 2012). The concatenated alignment consisted of 2287 base pairs (bp) including the five markers. All phylogenetic analysis were performed with MrBayes through the CIPRES science gateway (Miller et al. 2010). The parameters of the Bayesian analysis consisted of two independent runs of ten million generations each, with four Markov chains sampled every 1000 generations (mcmc ngen = 10000000 relburnin=yes burninfrac=0.25 printfreq=1000 samplefreq=1000 nchains=4 savebrlens=yes; sump relburnin=yes; sumt relburnin=yes; contype=halfcompat;). Tracer v. 1.6 (Rambaut 2018) was used to visualize parameter estimates and ensure that all estimates converged prior to removing a burnin period of 1 × 10^6^ generations. Convergence time among runs was determined as twice the number of generations it took the standard deviation of split frequencies to drop below 0.01.

## Results

### Species accounts

#### Tapinoma atriceps

Taxon classificationAnimaliaHymenopteraFormicidae

Emery, 1888

BC1E9755-A29D-51EF-ABC7-D294ED6A1857

**Figure 1.**
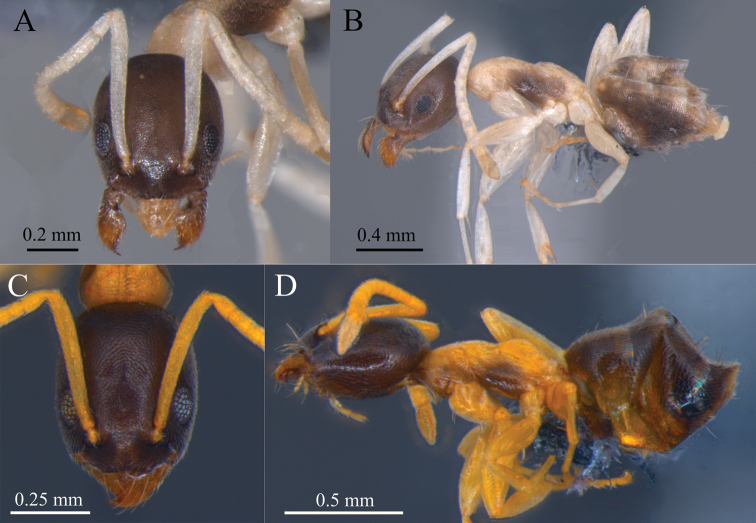
*Tapinoma atriceps* and *T. breviscapum* workers **A** head in frontal view of *T. atriceps***B** body in lateral view of *T. atriceps***C** head in frontal view of *T. breviscapum***D** body in lateral view of *T. breviscapum*. Photographed specimens and those included in the molecular analyzes (haplotypes H04 and H08 respectively) are nestmates. Specimens deposited in DZUP. Photographs by M. Escárraga.

**Figure 2.**
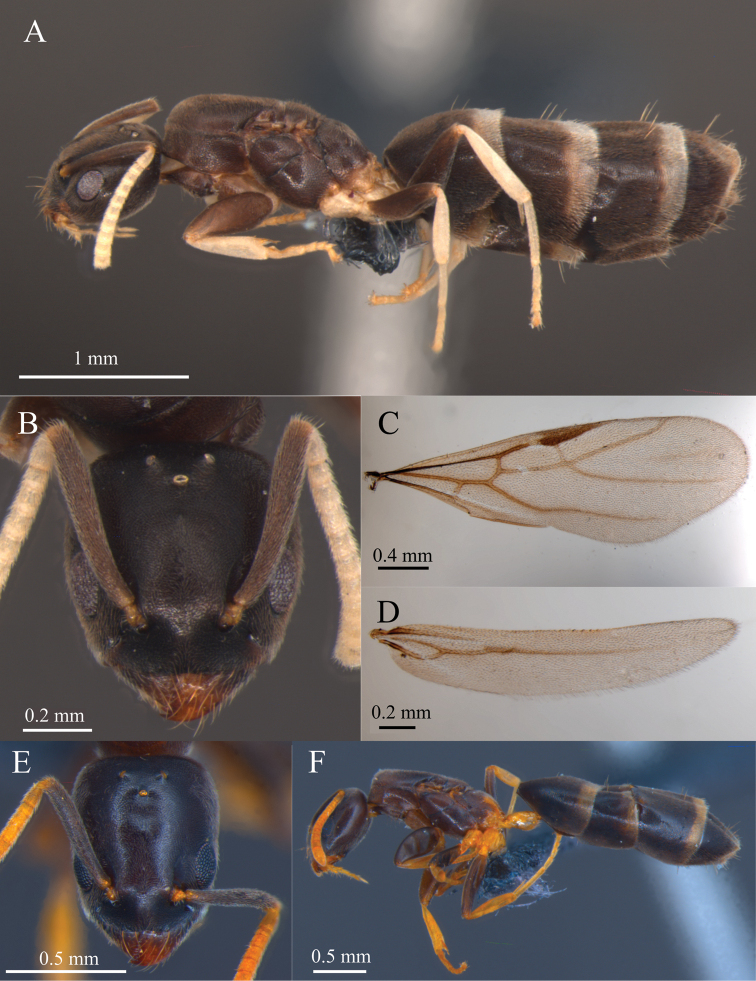
*Tapinoma atriceps* and *Tapinoma breviscapum* queen **A** body in lateral view of *T. atriceps***B** head in frontal view of *T. atriceps***C** anterior wing of *T. atriceps***D** posterior wing of *T. atriceps***E** head in frontal view of *T. breviscapum***F** body in lateral view of *T. breviscapum*. Photographed specimens and those included in the molecular analyzes (haplotypes H04 and H08 respectively) are nestmates. Specimens deposited in DZUP. Photographs by M. Escárraga.

**Figure 3.**
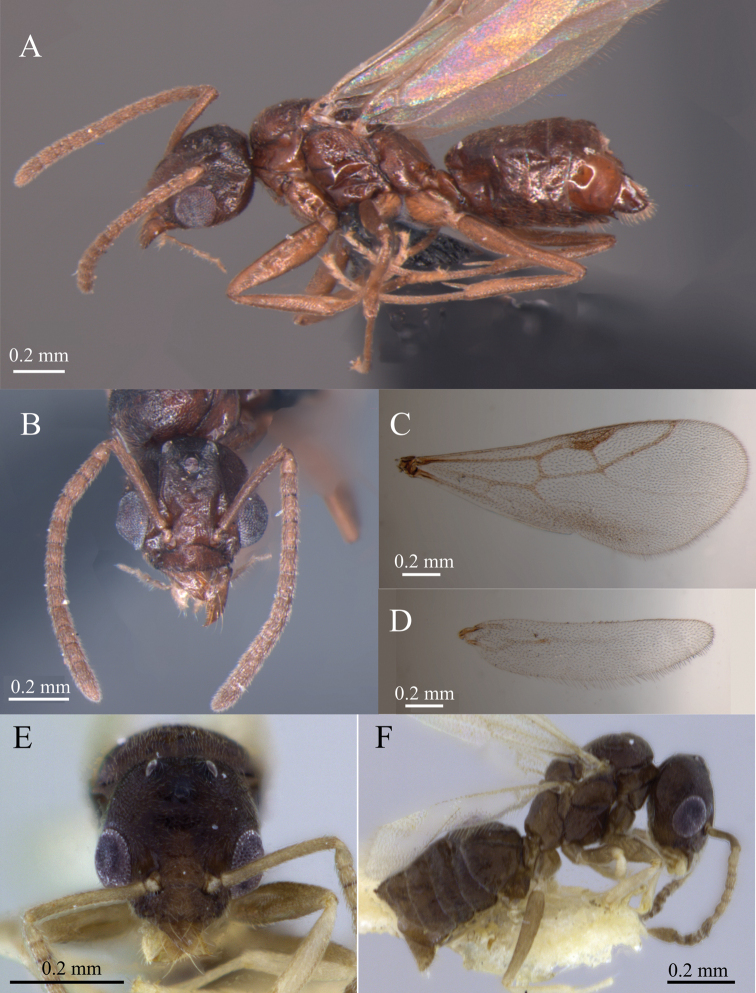
*Tapinoma atriceps* from Antonina, Reserva Natural Guaricica (Paraná, Brazil) and *T. breviscapum* from Serra do Cipó (Minas Gerais, Brazil) male **A** habitus of *T. atriceps***B** head in frontal view of *T. atriceps***C** anterior wing of *T. atriceps***D** posterior wing of *T. atriceps***E** head in frontal view of *T. breviscapum***F** habitus of *T. breviscapum*. Specimens deposited in DZUP. Photographs by M. Escárraga.

**Figure 4.**
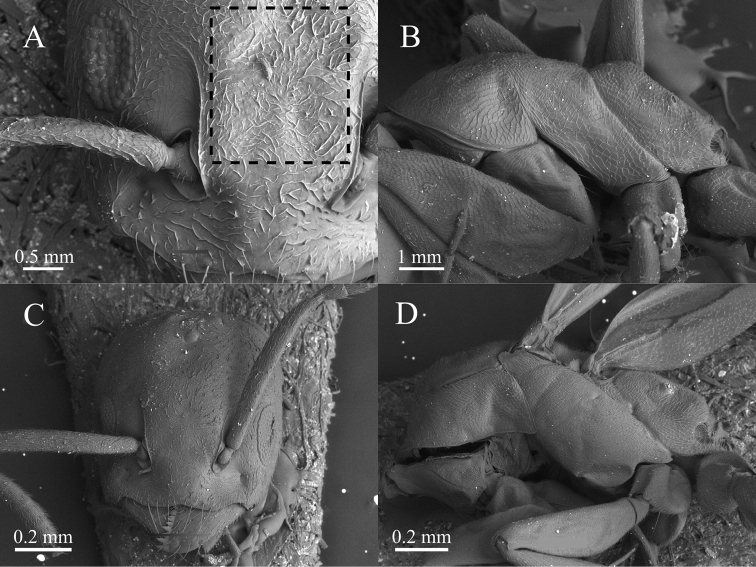
SEM microphotographs of *Tapinoma atriceps* from Serra do Cipó (Minas Gerais, Brazil) **A** worker head in dorsal view **B** worker mesosoma in lateral view **C** queen head in dorsal view **D** queen mesosoma in lateral view. The box with dashed lines indicates pubescence on the frons between the frontal carinae. Specimens deposited in DZUP.

**Figure 5.**
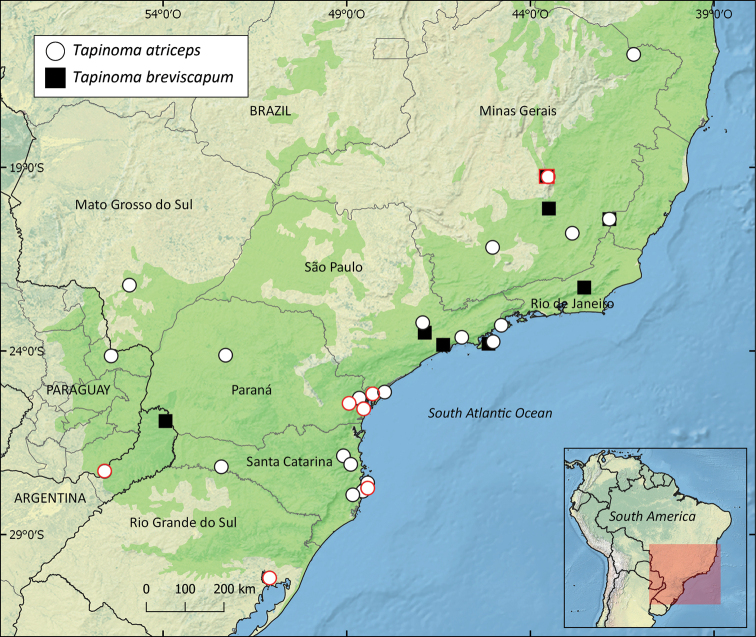
Distribution map of *Tapinoma atriceps* and *T. breviscapum*. The figures with red outline correspond to the localities for the sequenced specimens. Dark green area in the main map corresponds to the Atlantic Forest domain defined by the WWF.

Figs 1A, B , 2A, D , 3A, D , 4 , 5

Tapinoma (Micromyrma) atriceps Emery, 1888: 363. Syntype series (several workers, queens, males): Brazil, Rio Grande do Sul (v. Ihering) [MSNG, AntWeb image of syntype examined]. One syntype worker (CASENT0904029) here designated lectotype.
Tapinoma atriceps Emery. Kempf 1972: 247.
Tapinoma atriceps Emery. Shattuck 1994: 142.
Tapinoma atriceps Emery. Bolton 2021: e-catalogue (http://antcat.org).

##### Worker diagnosis.

Lateral margin of head in frontal view distinctly convex. Compound eye with 9 or 10 ommatidia along maximum diameter. Scape long (SI > 93). In profile, dorsal margin of propodeum forms distinct angle with propodeal declivity; dorsal margin short, about 1/4 length of declivitous margin (Fig. 4B).

*Worker*. Measurements (*n* = 26): HL 0.58 ± 0.04 (0.52–0.64), HW 0.50 ± 0.03 (0.42–0.55), SL 0.57 ± 0.04 (0.50–0.63), WL 0.69 ± 0.06 (0.60–0.78). Indices: CI 86 ± 3 (78–91), SI 97 ± 3 (93–103).

Head in full-face view oval, longer than wide, lateral margin convex, posterior margin slightly convex to straight (Fig. 1A). Maxillary palp relatively filiform, long, extending posteriorly beyond half of head. Masticatory margin of mandible with one large apical tooth, followed by two smaller teeth, fourth tooth larger than third, and then followed by denticles. Anterior margin of clypeus slightly emarginate medially. Scape almost as long as HL or greater (SI >93), surpassing posterior margin of head by distance equal to or greater than pedicel. In lateral view, dorsal margin continuously convex; metanotal groove weakly impressed; propodeum in lateral view slightly below level of mesonotum. Integument weakly imbricate, with exception of smooth petiole. Body covered by short, appressed pubescence. Head (excluding clypeus), antenna, and mesosoma lacking erect setae; clypeus with 6 long setae. Gastric tergites bearing erect hairs near their posterior margins: 2 hairs on first tergite, 2–4 on second, 4–6 on third, and 6–10 on fourth. Head and gaster medium brown; antennae, mesosoma, legs and petiole whitish yellow; mesosoma with brown spot on mesopleuron, spot sometimes present on lateroposterior corners of pronotum, metapleuron, and sides of propodeum.

*Queen*. Measurements (*n* = 4): HL 0.73 ± 0.03 (0.70–0.76), HW 0.68 ± 0.04 (0.64–0.71), SL 0.60 ± 0.02 (0.58–0.62), WL 1.23 ± 0.13 (1.08–1.32). Indices: CI 94 ± 3 (91–97), SI 83 ± 1 (82–83).

Head subquadrate in full-face view, slightly longer than broad (CI 91–97), lateral margin very convex, posterior margin straight to slightly convex (Fig. 2B). Mandibular masticatory margin with one large apical tooth, followed by 2 smaller teeth, fourth tooth larger than third, followed by 5 smaller teeth, and then small denticles decreasing in size. Anterior margin of clypeus slightly emarginate medially. Scape relatively long, reaching or surpassing posterior margin of head by length shorter than that of pedicel (SI 82–83). Forewing with crossveins 2r-rs, 2rs-m, and cu-a present (Fig. 2C, D). Hindwing with cu-a present, cubitus short, not projected after 1rs-m+M. Integument weakly imbricate; mesopleuron smooth. Body covered by short yellowish pilosity, excepting glabrous petiole. Clypeus bearing 6 long setae; gastric tergites each bearing several erect setae near their posterior margins. Body color medium brown; palps, flagella, coxae, trochanter, tibiae, tarsi, and petiole whitish yellow; propodeum usually brown, but sometimes with whitish-yellow spot on posterodorsal region. Gastric tergites I–III with pale-yellow, posterior transverse strip.

*Male*. Measurements (*n* = 4): HL 0.48 ± 0.04 (0.44–0.51), HW 0.52 ± 0.02 (0.50–0.53), SL 0.36 ± 0.04 (0.32–0.41), WL 0.63 ± 0.02 (0.61–0.66). Indices: CI 105 ± 11 (92–114), SI 76 ± 5 (71–82).

Head rounded in dorsal view; posterior margin slightly interrupted by posterior ocelli; anterior margin of clypeus straight to weakly emarginate medially. Eye large, rounded. Scape long, reaching or surpassing posterior head margin. Mandible semi-falcate; masticatory margin with large apical tooth followed by denticles of similar size forming a serrated surface continuing indistinctly up to the mandibular basal margin. Integument feebly imbricate, katepisternum smooth. On forewing, 1m-cu absent, median short. On hindwing, free section of radial and cu-a present, free section of cubitus absent. Row of long setae present on posterior margin of fore and hindwings. Head, scutum, and gaster covered by moderate, yellow, short, appressed hairs; scutellum glabrous. Hairs absent to scarce on pronotum, mesopleuron, propodeum, and petiole. Antenna covered by short, decumbent hairs. Gastric tergites I–V lacking erect setae. Head, mesosoma, petiole, and gaster dark brown. Antenna and legs light brown.

##### Distribution.

*Tapinoma atriceps* occurs in Argentina, Brazil, and Paraguay (Fig. 5). In Argentina, this species is present in the northeastern corner of the country, in the province of Misiones. In Brazil, our records show the presence of this species in the states of Mato Grosso do Sul, Minas Gerais, Paraná, Rio Grande do Sul, Santa Catarina, and São Paulo. In Paraguay, *T. atriceps* occurs in the department of Canindeyú.

##### Biology.

*Tapinoma atriceps* is an arboreal ant which can be found from the understory layer to the canopy and rarely on the ground. We found nests of this ant in hollow cavities of the vegetation or dry hanging branches, in plants of the families Poaceae (Bambusoideae), Melastomataceae, Piperaceae, and Urticaceae. Workers are commonly found foraging on the leaves of plants near the nest. The colony can be moderately large, with more than 312 workers, and in a couple of nests we found four dealate queens, evidencing polygyny as in other species of *Tapinoma* (e.g., Bustos and Cherix 1998; Buczkowski and Bennet 2008).

##### Material examined.

Argentina • 2 queens, 2 workers; Misiones, Parque Provincial Teyú Cuaré; 27°17.08'S, 55°35.62'W; 28 Dec. 2007; W. Mackay and E. Mackay legs; WEMC. BRAZIL • 1 worker; Mato Grosso do Sul, Dourados, Fazenda Azulão; 22°12.800'S, 54°55.133'W; 10 Mar. 2006; M. Santana and A.Vieira legs; DZUP • 2 males, 4 workers; Minas Gerais, Alto Caparaó, Parque Nacional Caparaó, 20°25.155'S, 41°51.083'W; 5–20 Dec. 2011; J. Chaul leg. DZUP • 1 worker; Minas Gerais, Lavras, Fragmento 06; Dec. 2002; M.S. Santos and N.S. Dias legs; CEPLAC • 1 queen; Minas Gerais, Pedra Azul; alt. 800 m; Nov. 1972, Seabra and Alvarenga legs; MZSP 10657 • 2 workers; Minas Gerais, Pedra Azul, Seabra and Alvarenga legs; MZSP 10658 • 1 worker, 1 queen; Minas Gerais, Serra do Cipó, 19°25.155'S, 43°51.083'W; 26 Ju. 2016; F. Siqueira leg.; DZUP • 1 worker; Minas Gerais, Viçosa, Mata do Paraíso; 1997/1998; S. de M. Soares leg.; CEPLAC • 1 male, 1 queen, 1 worker; Paraná, Antonina, Reserva Natural Guaricica, 25°17.794'S, 48°39.592'W; 26 Dec. 2016; C. da Costa leg.; DZUP • 1 queen, 1 worker; Paraná, Antonina, Reserva Natural Guaricica; 25°18.354'S, 48°39.678W; 29 Oct. 2016; M. Escárraga leg.; DZUP 548798 • 1 worker; Paraná, Antonina, Reserva Natural Guaricica; 10–13 Jul. 2016; M. Escárraga leg.; DZUP 548797 • 2 workers; Paraná, Guaraqueçaba, Reserva Natural Salto Morato, 25°09.816'S, 48°17.880'W; 8 Dec. 2016; M. Escárraga leg.; DZUP 548784 • 1 worker; Paraná, Paranaguá, Parque Estadual do Palmito; 25°35.016'S, 48°32.496'W, 28 Apr. 2016; M. Escárraga leg.; DZUP 548786 • 2 workers; Paraná, Pq. Marumby, Km 34, Estr. Graciosa; 3 Oct. 1980; A.L. Lozovei leg.; MZSP14069 • 1 worker; Rio Grande do Sul; MZSP11439 • 1 worker; Rio Grande do Sul, Porto Alegre, Morro São Pedro, 30°10.824'S, 51°06.078'W; L. Kaminski leg.; DZUP • 2 workers; Rio Grande do Sul, Porto Alegre; 30°10.824'S, 51°06.078'W; 27 Dec. 2016; M. Escárraga leg.; DZUP 548788 • 3 workers; Santa Catarina, Blumenau; alt. 120 m;19 Jan. 1972; W.W. Kempf leg.; MZSP 7049 • 1 worker; Santa Catarina, Brusque, RPPN Chácara Edith; 27°05.692'S, 48°53.581'W; 28 Feb. 2013; Y. Gadelha leg.; DZUP • 1 worker; Santa Catarina, Florianópolis, Naufragados; 27°49.405'S, 48°33.694'W; 19 Feb. 2016; J. Chaul leg.; DZUP • 2 workers; Santa Catarina, Florianópolis; 27°35.928'S, 48°25.962'W; 25 Feb. 2017; M. Escárraga leg.; DZUP 548789 • 1 worker; Santa Catarina, Florianópolis, Praia Mole, 27°35.927'S, 48°25.962'W; 25 Feb. 2017; A. Menezes leg.; DZUP • 2 workers; Santa Catarina, Seara, Nova Teutônia; Jul. 1959; F. Plaumann leg.; MZSP • 1 worker; Santa Catarina, Seara, Nova Teutônia; 19 Dec. 1972; F. Plaumann leg.; MZSP8566 • 2 workers; Santa Catarina, Palhoça, Parque Estadual da Serra do Tabuleiro; 27°44.467'S, 48°41.833'W; 2–10 Jun. 2003; winkler 30; R.R. Silva, B. H. Dietz and A. Tavares legs.; MZSP • 3 workers; Santa Catarina, Seara, 24°07'S, 52°18'W; Jul. 1999; R. R. Silva leg.; Transecto II Isca Veget.; MZSP • 1 worker; Santa Catarina, Seara; Jul. 1958; F. Plaumann leg.; MZSP 2719 • 1 worker; Santa Catarina, Santo Amaro da Imperatriz, Parque Estadual da Serra do Tabuleiro, 27°55.356'S, 48° 50.277'W; 26 Nov. 2013; Y. Gadelha leg.; DZUP • 1 male, 1 queen, 1 worker; São Paulo, 8 Km SW Jundiaí; 23°14'S, 46°56'W; alt. 1180m,; 28 Dec. 1993; Manual; P.S. Ward leg.; [PSWC 12463] • 2 queens, 3 workers; São Paulo, Ilha da Vitória, 29 Mar.–6. Apr. 1965; Exp. Depto. Zool. legs; MZSP 4083 • 1 queen; São Paulo, Ilha da Vitória; 16–27 Mar. 1964; Exp. Depto. Zool. leg.; MZSP 4176 • 3 workers; São Paulo, Ilha da Vitória; 29 Mar.–6 Apr. 1966; Exp. Depto. Zool. leg.; MZSP 4117; • 2 workers; São Paulo, Ilha do Cardoso; Jan. 1979; Liliana Foneris leg.; MZSP • 2 workers; São Paulo, Salesópolis, Est. Biol. Boracéia; 11 Nov. 1960; K. Lenko leg.; MZSP 1788 • 4 workers; São Paulo, Salesópolis, Est. Biol. Boracéia; 13 Nov. 1960; K. Lenko leg.; MZSP 1483 • 1 worker; São Paulo, Salesópolis, Est.Biol. Boracéia, 3–5 May 1996; Brandão, Agosti, Diniz, Silvestre and Yamamoto legs; MZSP • 6 workers;São Paulo; USNM • 1 worker; São Paulo, Ubatuba, Parque Estadual Serra do Mar; 23°17.940'S, 44°47.220'W; 3–14 Mar. 2008; F. Esteves and R. Feitosa legs; MZSP • PARAGUAY. 1 worker; Canindeyú, Res. Nat. Bosque Mbaracayú, Jejuimí; alt. 107 m; 28 May–5 Jul.1996; Malaise; A.C.F. Costa leg.; ALWC.

#### Tapinoma breviscapum

Taxon classificationAnimaliaHymenopteraFormicidae

Forel, 1908 status novo

665E07B2-A66F-5E0B-B602-16F660FE11B3

**Figure 6.**
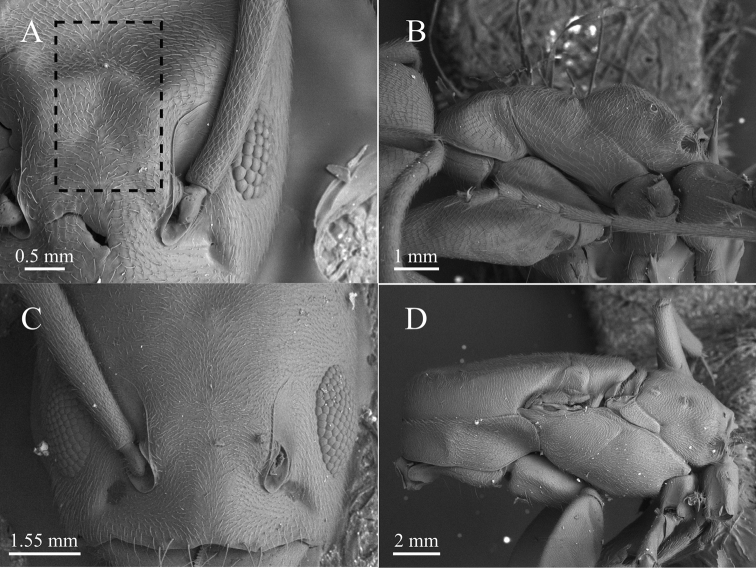
SEM microphotographs of *Tapinoma breviscapum* from Serra do Cipó (Minas Gerais, Brazil) **A** worker head in dorsal view **B** worker mesosoma in lateral view **C** queen head in dorsal view **D** queen mesosoma in lateral view. The inset with broken lines indicates finer and lesser separated pubescence on the frons between the frontal carinae. Specimens deposited in DZUP.

Figs 1C, D , 2E, F , 3E, F , 5 , 6

Tapinoma atriceps breviscapa Forel, 1908: 384–385. Syntype series (worker, queen): Brazil, São Paulo, Raiz da Serra. [MHNG, examined]. One syntype worker (CASENT0909768) here designated lectotype; two workers designated as paralectotypes, uppermost specimen and lowermost on the same pin as lectotype worker (MHNG, examined).
Tapinoma atriceps breviscapum Forel. Kempf 1972: 247.
Tapinoma atriceps breviscapum Forel. Shattuck 1994: 142.
Tapinoma atriceps breviscapum Forel. Bolton 2021: e-catalogue (http://antcat.org).

##### Worker diagnosis.

Lateral margin of head in frontal view slightly convex. Eye with 7 or 8 ommatidia along maximum diameter. Scape short (SI < 85). Dorsal face of propodeum in lateral view meeting propodeal declivity through rounded angle; length of dorsal margin about ½ that of declivity.

*Worker*. Measurements (*n* = 16): HL 0.58 ± 0.04 (0.53–0.66), HW 0.48 ± 0.04 (0.43–0.56), SL 0.48 ± 0.03 (0.44–0.56), WL 0.63 ± 0.06 (0.49–0.78). Indices: CI 83 ± 1 (82–85), SI 80 ± 2 (78–85).

Head in full-face view oval to rectangular, longer than wide; lateral and posterior margins slightly convex. Maxillary palps filiform, relatively short, not posteriorly surpassing beyond mid-length of head. Mandibles with masticatory margin with 1 large apical tooth, followed by 2 smaller teeth, fourth tooth larger than third, and then followed by denticles. Anterior margin of clypeus slightly emarginate medially. Scape relatively short when compared to *T. atriceps* (SI < 85), reaching or surpassing posterior margin of head by distance shorter than pedicel length. Pronotum and mesonotum form continuous feeble convexity in lateral view; metanotal groove weakly impressed; propodeum dome-shaped, slightly higher than mesonotum. Integument weakly imbricate, excepting petiole which is smooth. Body covered with short decumbent pubescence. Head (excluding clypeus), antenna, and mesosoma lacking erect setae, clypeus with 6 anterior setae. Pilosity pattern on gastric tergites similar as to *T. atriceps*. Head and gaster medium brown; antenna, mesosoma, legs, and petiole pale whitish yellow to bright orange (Fig. 1C, D). Mesosoma with brown spot on mesopleuron and lateral pronotum, sometimes present on metapleuron and lateral propodeum, almost completely covering mesosomal side.

*Queen*. Measurements (*n* = 6): HL 0.74 ± 0.04 (0.70–0.80), HW 0.64 ± 0.03 (0.59–0.68), SL 0.54 ± 0.04 (0.49–0.59), WL 1.24 ± 0.18 (1.07–1.46). Indices: CI 86 ± 1 (84–87), SI 73 ± 2 (70–76).

Head rectangular in full-face view, clearly longer than wide (CI 84–87); lateral and posterior margins straight. Masticatory margin of mandible with 1 large apical tooth, followed by 2 smaller teeth, fourth tooth larger than third, followed by 5 smaller teeth, and then small denticles decreasing in size. Clypeus slightly emarginate anteromedially. Scape short, never surpassing posterior margin of head (SI 72–76). Integument weakly imbricate, mesopleuron smooth. Dorsum of head with abundant, short, decumbent hairs; clypeus bearing 6 long hairs; gastric tergites with several erect setae near their posterior margins. Body medium brown; palps, flagellum, coxae, trochanters, tibiae, tarsi, and petiole whitish yellow to bright orange (Fig. 2E, F); propodeum sometimes with whitish-yellow spot on posterodorsal region. Gastric tergites I–III with pale-yellow, transverse posterior strip.

*Male*. Measurements (*n* = 3): HL 0.46 ± 0.02 (0.43–0.48) HW 0.46 ± 0.03 (0.43–0.48) SL 0.36 ± 0.02 (0.33–0.38) WL 0.62 ± 0.02 (0.61–0.64). Indices: CI 99 ± 2 (97–100) SI 78 ± 3 (75–82)

Head in dorsal view rounded, posterior margin slightly interrupted by lateral ocelli; anteromedian margin of clypeus straight to weakly emarginate. Compound eye large, rounded; scape long, reaching posterior margin of head; maxillary palp filiform. Mandible semi-falcate; masticatory margin with large apical tooth followed by many teeth of similar size. Integument feebly imbricate, katepisternum smooth. Forewing with median short; hindwing with free section of radial and cu-a present, free section of cubitus absent. Row of long setae present on posterior margins of fore and hindwing. Head, scutum, scutellum, and gaster covered by moderately abundant, yellow, short, decumbent hairs; antenna covered by short, decumbent hairs. Anepisternum covered by hairs, katepisternum lacking hairs ventrally. Gastric tergites I–V lacking erect setae. Head, mesosoma, petiole, and gaster dark brown; antenna and legs light brown.

##### Distribution.

*Tapinoma breviscapum* has been recorded from Misiones, Argentina, and from the Brazilian states of Minas Gerais, Rio de Janeiro, and São Paulo (Fig. 5).

##### Biology.

*Tapinoma breviscapum* is an arboreal ant, but beyond that, there is not much available information. This species, reported as *T. atriceps*, was found inhabiting a gall of *Microgramma squamulosa* (Kaulf.) de la Sota (Santos et al. 2019).

##### Material examined.

Argentina • 3 workers; Misiones, 40.66K N Campinas de América; 25°55.153'S, 53°56.151'W; alt. 508 m; 3 Jan. 2008; W. Mackay and E. Mackay legs; WEMC#22811 BRAZIL • 2 queens, 1 worker; Minas Gerais, Alto Caparaó, Parque Nacional Caparaó; 20°25.155'S, 41°51.083'W; 5-20 Dec. 2011; J. Chaul leg.; DZUP • 2 males, 3 workers; Minas Gerais, Serra Caraça; 1380 m; Nov. 1961; K. Lenko, Martins and Silva legs; MZSP 3104 • 3 workers; Minas Gerais, Serra Caraça; 1380m; Nov. 1961; K. Lenko, Martins and Silva legs; MZSP 4216 • 1 male, 2 queens, 17 workers; Minas Gerais, Serra do Cipó, CAPIII; Jan. 2013; M. Anjos M. leg.; DZUP • 1 queen, 2 workers; Minas Gerais, Serra do Cipó, Capão 11; 19°14.873'S, 43°33.055'W; 25 Jun. 2017; H. Brant leg. DZUP • 5 workers; Minas Gerais, Serra do Cipó; 19°14.874'S, 43°33.054'W; 25 Jun. 2017; M. Escárraga leg., MYR380; Genbak codes: MG920285, MN294973, MN294963, MT375619; DZUP 548800 • 1 worker; Rio de Janeiro, Nova Friburgo, Praça do Suspiro, 22°16.763'S, 42°32.148'W; Apr. 2016-Feb. 2018; I. Lancelloti leg.; DZUP • 5 workers; São Paulo, Barueri; K. Lenko leg.; MZSP 447 • 5 workers; São Paulo, Barueri; K. Lenko leg.; MZSP 2477 • 5 workers; São Paulo, Barueri; 19 Jul. 1958; K. Lenko leg.; MZSP 534 • 3 workers; São Paulo, Cubatão, Estação Raiz da Serra; v Ihering leg.; MHNG • 2 queens, 2 workers; São Paulo, Ilha dos Búzios, 2 Apr. 1964; Exp. Dep. Zool. legs; MZSP 3910 • 1 male, 1 queen, 2 workers; São Paulo, Ilha dos Búzios, 2 Apr. 1964, Exp. Dep. Zool. legs; MZSP 4105 • 2 workers; São Paulo, Ilha dos Búzios; 3 Sep. 1964; Exp. Dep. Zool. legs; MZSP 3885 • 4 workers; São Paulo, Ilha dos Búzios; 3 Apr. 1964; Exp. Dep. Zool. legs; MZSP 3885 • 1 male, 1 queen, 3 workers; São Paulo, Ilha dos Búzios, 19 Oct. 1963; Exp. Dep. Zool. legs; MZSP 2978 • 1 queen, 4 workers; São Paulo, Ilha dos Búzios; 17 Oct. 1963; Exp. Dep. Zool. leg; MZSP 2994 • 1 queen, 4 workers; São Paulo, Ilha dos Búzios; 26 Oct. 1963; Exp. Dep. Zool. legs; MZSP 2992 • 1 queen, 3 workers; São Paulo, Ilha dos Búzios; 31 Jul. 1964; Exp. Dep. Zool. legs; MZSP 3616 • 1 male, 1 queen, 2 workers; São Paulo, Ilha dos Buzios; 2 Apr. 1964; Exp. Dep. Zool. [MZSP 4105] • 1 worker; São Paulo; MZSP11974 • 6 workers; São Paulo; USNM.

###### Morphological separation between *Tapinoma atriceps* and *T. breviscapum*

**Figure 7.**
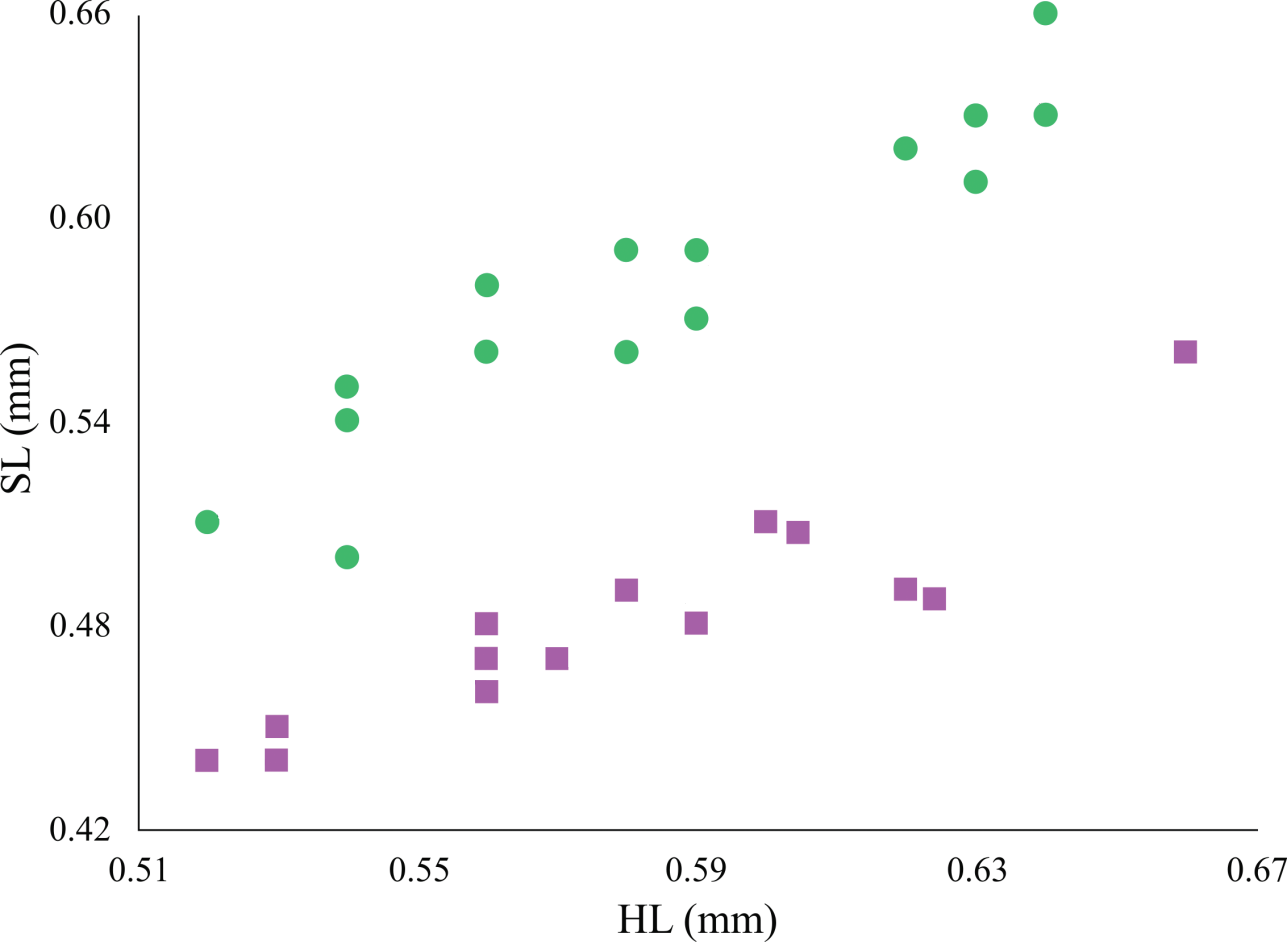
Morphometric scatterplot showing the relationship between HL and SL. The green circles correspond to the measurements of *Tapinoma atriceps*, while the purple squares correspond to those in *T. breviscapum*.

The most readily recognizable morphological diagnostic traits that permit separation of *T. atriceps* and *T. breviscapum* workers and queens are the relative length of the scape (i.e., SI), the shape of the propodeum, and differences in the degree of cephalic pubescence. In *T. atriceps* the worker scape is almost as long as the HL or greater (SI >93; Fig. 1A), in contrast with *T. breviscapum*, where it is relatively short (SI < 85; Fig. 1B), sometimes reaching or barely surpassing the posterior head margin by a distance shorter than the pedicel length. SL shows significant differences between the workers of each species (*T* = 7.51, *p* < 0.0001). Although there is a certain degree of overlap in the absolute measure (0.50–0.63 in *T. atriceps* and 0.44–0.56 in *T. breviscapum*) the relationship from SL to HL for each species showed non-overlapping ranges (Fig. 7). Other morphometric traits, such as HL, HW, and WL were also evaluated; however, each of their paired distributions overlapped, showing no statistical differences. The SL partially overlaps in queens of both species (0.58–0.62 in *T. atriceps* and 0.49–0.59 in *T. breviscapum*); however, differences between species were found (*T* = 2.29, *p* = 0.0257). These differences are notable in the non-overlapping ranges of the relative length of the scape (82–83 and 70–76, respectively). Statistical differences were also found in the HW of both species (*T* = 2.26, *p* = 0.0268); even without measuring, these differences are evident when they are compared under a stereoscope (Fig. 2B vs 2E), as *T. breviscapum* queens have a more elongate head as reflected in CI values that do not overlap those of *T. atriceps* queens.

The worker propodeum in both species differs markedly in shape and in the proportions between the dorsal and the posterior faces (Fig. 4B vs 6B). The dorsal propodeal margin when seen laterally in *T. atriceps* forms a distinct blunt angle with the declivity, contrasting with the rounded convexity formed in *T. breviscapum*. Additionally, the dorsal margin in *T. atriceps* is about 1/4 the length of the declivitous margin, while in *T. breviscapum* it is longer, about 1/2 that of the declivity (Figs 1B, D, 4B, 6B). The dorsal surface of the head in *T. atriceps* workers (Fig. 4A) is covered by appressed pubescence that is relatively longer and sparser than in *T. breviscapum*, where it is abundant and relatively shorter (Fig. 6A). The males of both species are relatively similar in morphology(Fig. 3), but the male of *T. breviscapum* can be differentiated from *T. atriceps* males because the former is on average slightly larger (0.63 ± 0.02 mm) and the scutellum is glabrous, while males of *T. breviscapum* are slightly smaller (0.62 ± 0.02 mm) and have decumbent hairs on the scutellum.

###### Genetic differentiation between *Tapinoma atriceps* and *T. breviscapum*

Final alignments had 648, 562, 422, and 655 bp for COI, LW Rh, Wg, and EPIC, respectively. For LW Rh, the length of the concatenated two flanking exonic sequences was 456 bp and for the intron 106 bp.

The greatest genetic variation among the molecular markers was observed in COI, followed by EPIC, Wg, LW Rh-ex, and LW Rh-in, with 114, 38, 15, 9, and 3 variable sites, respectively. Within the *T. atriceps* samples, the genetic pairwise distance ranged between 0.0–9.6% for COI, 0.0–2.0% for LW Rh-intron and Wg, 0.0–1.8% for EPIC, and 0.0–0.4% for LW Rh-exon. The mean genetic distance between *T. atriceps* and *T. breviscapum* was 9.4% (8.8–10%) for COI. For the nuclear markers, EPIC presented the greatest distance between the two species (4.6–4.7%), followed by Wg (1.4–5.9%), LW Rh-intron (1.1–2.3%), and LWRh-exon (1.5–1.7%).

**Figure 8.**
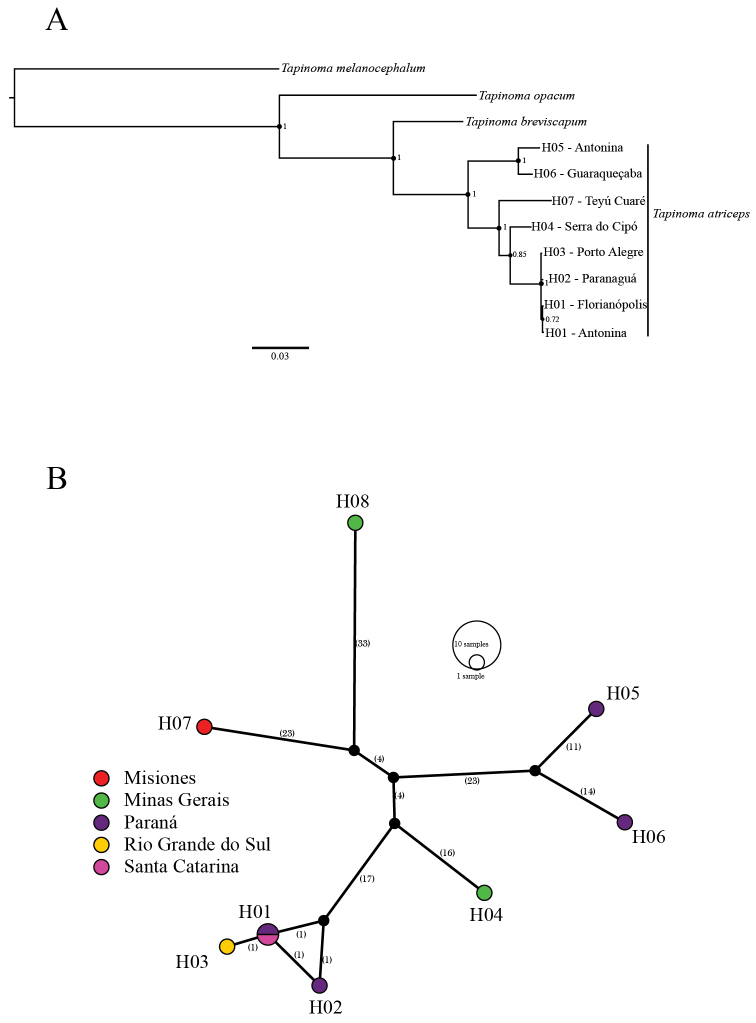
Evolutionary relationships between *Tapinoma atriceps* and *Tapinoma breviscapum***A** bayesian phylogenetic reconstruction based on concatenated sequences of four molecular markers (COI, LWRh, WG, and EPIC). The scale bar represents substitutions per site and the number next to nodes the posterior probability **B** haplotype network including several populations of *T. atriceps* and a single sample of *T. breviscapum* (H08). Every circle represents a different haplotype, the color corresponds to the geographical distribution, and the number between brackets corresponds to the number of mismatches between haplotypes.

In the phylogenetic reconstruction (Fig. 8A), *T. opacum* is closer to *T. atriceps* and *T. breviscapum* than to *T. melanocephalum*. *Tapinoma atriceps* was recovered as a monophyletic group, sister to *T. breviscapum*. The Bayesian consensus trees of the individual analysis of each locus also recover both results (Suppl. material 4: Figure S1). Bayesian analysis of COI provided a topology similar to the Bayesian tree based on concatenated data, although with differences in branch lengths and node support of *T. atriceps* (PP = 0.71). The reconstructed separate trees with the nuclear loci also recovered *T. breviscapum* as a sister species to *T. atriceps* (Suppl. material 4: Figure S1) but the latter results in poor resolution among the sampled populations. Within *T. atriceps*, the topology derived from the concatenated data was relatively similar to the haplotype network (Fig. 8B). The samples from Paraná, corresponding to haplotypes H05 and H06, presented a comparatively deep divergence from the rest of the species. The sample of *T. atriceps* from Minas Gerais was sister to the group from southern Brazil, the populations of this latter group showing little genetic structure. Most of the nodes were relatively well-supported (PP 0.85–1.00) except for the low value of support corresponding to the Antonina haplotype (H01).

Seven mitochondrial (COI) haplotypes of *Tapinoma atriceps* were identified for the eight analyzed sequences and a single mitochondrial haplotype for the only *T. breviscapum* sample (Fig. 8B). The analysis estimated five unsampled haplotypes and found many mismatches between most of the haplotypes, evidencing high molecular variation for this marker. Haplotype H01, found in Paraná and Santa Catarina, is very close to haplotypes H02 and H03 and separated from them by only one nucleotide substitution; together, these form a group of haplotypes from southern Brazil. Other two haplotypes found in Paraná (H05 and H06) are relatively close to each other, and separated by one unsampled haplotype, but very different from the other haplotypes from Paraná. Haplotype H04 from Minas Gerais is closest to the H01–H03 haplotype group, separated by two unsampled haplotypes. Haplotype H07 from Misiones (Argentina) is closer to *T. breviscapum* haplotype (H08) and relatively close to the subset of haplotypes from the southeastern (H04) and southern Brazil (H01–H03). A haplotype network built with only the sampled populations of *T. atriceps* (Suppl. material 5: Figure S2) shows that the H07 haplotype is sister to the other haplotypes from southeastern and southern Brazil, which is congruent with the phylogenetic structure inferred from the concatenated molecular data (Fig. 8A).

## Discussion

*Tapinoma atriceps* and *Tapinoma breviscapum* can be differentiated from other Neotropical *Tapinoma* ants by their particular bicolored pattern. Other *Tapinoma* can be mostly pale yellow or uniform brown, with yellow antennal scapes and coxae, but never with a spot on the mesopleuron, nor the bicolored pattern of *T. atriceps* and *T. breviscapum*. Only two other ant species that occur in South America, *Tapinoma melanocephalum* (Fabricius, 1793) and *Linepithema leucomelas* (Emery, 1894), have similar colors and size that could lead to confusion. In the case of *T. melanocephalum*, a common invasive species, the head and mesosoma is dark brown and the gaster is pale yellow (Guerrero 2018). *Linepithema leucomelas* can be differentiated by the characters that define the genus: presence of a well-developed petiolar scale and mandibular dentition which presents teeth alternating with denticles (Wild 2007b).

*Tapinoma atriceps* and *T. breviscapum* are typical representatives of the genus in the Atlantic Forest of southwestern Brazil. Because of their sympatric distribution and morphological similarity, it has been difficult to separate them and the name *T. atriceps* has prevailed. Before the present work, *T. breviscapum* was only known from the type locality, Raiz da Serra in São Paulo (Forel 1908). We found that this species has a broader distribution, occurring in other Brazilian states such as Minas Gerais and Rio de Janeiro, and as far south as Misiones, Argentina. Such a broad distribution raises questions about the geographical origin of both species, which must be analyzed through a robust phylogeny and biogeographic inference of the genus *Tapinoma* in the Neotropical region.

Morphologically, *T. atriceps* and *T. breviscapum* can be differentiated by metric features associated with the head of the worker and queen, while the shape of the propodeum and hairs on the head allows the separation between the workers of both species, but the color of the body of the workers and queens in both species is relatively similar (see taxonomic treatment). Although the latter is true when species are allopatrically distributed, when occurring sympatrically they may exhibit no overlap of this trait (i.e., perhaps evidencing character displacement). The coloration pattern of workers and queens of *T. atriceps* and *T. breviscapum* from Serra do Cipó (MG, Brazil), corresponding to haplotypes H04 and H08, respectively, contrasts notably: antenna, mesosoma, legs, and petiole pale whitish-yellow in *T. atriceps* (Figs 1A, B, 2A, B) while those same sclerites are bright orange in *T. breviscapum* (Figs 1C, D, 2E, F). Differences in color are also observed in the worker and queen of both species from Misiones (Argentina), but the same sclerites which are bright orange in *T. breviscapum* are paler when compared to the H08 haplotype (R. Guerrero personal observation). This contrasting coloration pattern in sympatry could have played a fundamental role in the separation of lineages by reinforcing reproductive barriers between *T. atriceps* and *T. breviscapum* populations. The integration of comparative morphological analyzes of the genitalia of the males in both species and the analysis of more molecular data are necessary to elucidate aspects related to this evolutionary hypothesis.

The molecular analyses, including both mitochondrial and nuclear data, support the monophyly of *T. atriceps* (Fig. 8A), but we could not assess the monophyly of *T. breviscapum* because we could only analyze one sample of this species. The preliminary phylogenetic results of a broader study of *Tapinoma*, which includes several samples of both species from Minas Gerais and Misiones, confirm the reciprocal monophyly between them (R.J. Guerrero unpublished data). The COI-based Bayesian tree (Suppl. material 4: Figure S1) and the mitochondrial haplotype network (Fig. 8B) are very similar to the concatenated Bayesian tree, showing only minor differences in the position of the Misiones haplotype (H07) within *T. atriceps*. The other molecular markers also recovered *T. breviscapum* as sister to *T. atriceps* but failed to establish relationships among *T. atriceps* populations. Congruence between COI and the other nuclear markers is likely to be the result of similar differential lineage sorting. Although the Bayesian trees of COI and EPIC (Suppl. material 4: Figure S1) result in topologies with consistently different branch lengths, both markers show a similar phylogenetic relationship pattern within *T. atriceps*.

The average genetic distance between *T. breviscapum* and *T. atriceps* using COI (9.4%) is relatively high when compared with other *Tapinoma* species. For instance, Seifert et al. (2017) found genetic distances varying between 1.8% and 4.8% for pairs of *Tapinoma* species from the Mediterranean region using the same marker, considerably smaller values than those found in this study. The intraspecific variation in COI for *T. atriceps* is also considerably high (maximum of 9.6%) in comparison with *T. ibericum* Santschi, which has a distance of 1.3% as the greatest intraspecific variation (Seifert et al. 2017).

The highest values of intraspecific genetic distance in *Tapinoma atriceps* (9.6%) overlap with those between *T. breviscapum* and *T. atriceps* (8.8–10%); however, the greatest variation within *T. atriceps* species was found by comparing two samples from Paraná (H05–H06) with the rest of *T. atriceps* populations (Fig. 8B, Suppl. material 5: Figure S2). Such high genetic distance suggests the existence of cryptic diversity within this taxon, perhaps as the result of past climatic changes in the southern Atlantic Forest (Ströher et al. 2019), but divergence times estimates are necessary to obtain an approximation to this remarkable intrapopulation genetic differentiation. Despite the cryptic diversity suggested by COI we did not find any distinct morphological character in the workers and queens from Paraná (H05–H06) that would allow them to be separated from other *T. atriceps* specimens; therefore, we suggest these populations as part of the metapopulation of *T. atriceps* distributed in the Brazilian Atlantic Forest. Morphological analysis of more specimens, mainly males, in a wider geographical sampling throughout the Atlantic Forest biome could shed light on the intrapopulation mitochondrial genetic variation found in *T. atriceps*.

## Conclusions

We found that a native *Tapinoma* occurring in the Atlantic Forest and previously considered as different phenotypes of the same species, correspond in fact to two different species, *Tapinoma atriceps* and *Tapinoma breviscapum*, based on morphological and molecular evidence. We also found high COI variation within *T. atriceps* populations, suggestive of cryptic diversity. However, these results should continue to be explored with a broader sampling, as more population samples might be needed to understand phylogeographic patterns in *T. atriceps* and *T. breviscapum*. Additionally, those phylogeographic patterns could help in understanding the biogeographic history of the Atlantic Forest. Finally, a complete phylogenetic framework is needed to understand the origin and evolution of *Tapinoma* in the Neotropical region.

## Supplementary Material

XML Treatment for Tapinoma atriceps

XML Treatment for Tapinoma breviscapum

## Supplementary materials

Supplementary material 1 Table S1. Results of Normality test of the data in workers, queens, and males of *Tapinoma atriceps* and *T. breviscapum* Data type statistical data Explanation note *n* = number of specimens included in the analysis. SD = Standard deviation. W= Shapiro–Wilk test. *p* = probability associated with the distribution of the statistical test W. * indicates those morphometric characters that showed normality. https://binary.pensoft.net/file/534738This dataset is made available under the Open Database License (http://opendatacommons.org/licenses/odbl/1.0/). The Open Database License (ODbL) is a license agreement intended to allow users to freely share, modify, and use this Dataset while maintaining this same freedom for others, provided that the original source and author(s) are credited.Mayron E. Escárraga, John E. Lattke, Marcio R. Pie, Roberto J. Guerrero

Supplementary material 2 Table S2. Genbank codes of DNA sequences of *Tapinoma melanocephalum* and *T. opacum*, downloaded from Genbank Data type molecular data https://binary.pensoft.net/file/534739This dataset is made available under the Open Database License (http://opendatacommons.org/licenses/odbl/1.0/). The Open Database License (ODbL) is a license agreement intended to allow users to freely share, modify, and use this Dataset while maintaining this same freedom for others, provided that the original source and author(s) are credited.Mayron E. Escárraga, John E. Lattke, Marcio R. Pie, Roberto J. Guerrero

Supplementary material 3 Table S3. Substitution models selected with PartitionFinder v. 2.1 for each genetic marker Data type molecular data https://binary.pensoft.net/file/534740This dataset is made available under the Open Database License (http://opendatacommons.org/licenses/odbl/1.0/). The Open Database License (ODbL) is a license agreement intended to allow users to freely share, modify, and use this Dataset while maintaining this same freedom for others, provided that the original source and author(s) are credited.Mayron E. Escárraga, John E. Lattke, Marcio R. Pie, Roberto J. Guerrero

Supplementary material 4 Figure S1. Bayesian consensus trees from analyses of individual loci. Data type phylogenetic tree Explanation note Nodes with posterior probabilities greater than 80% are marked with circles. Posterior probabilities less than 80% are included. The scale bar represents substitutions per site and the number next to nodes the posterior probability. Outgroups have been pruned. https://binary.pensoft.net/file/534741This dataset is made available under the Open Database License (http://opendatacommons.org/licenses/odbl/1.0/). The Open Database License (ODbL) is a license agreement intended to allow users to freely share, modify, and use this Dataset while maintaining this same freedom for others, provided that the original source and author(s) are credited.Mayron E. Escárraga, John E. Lattke, Marcio R. Pie, Roberto J. Guerrero

Supplementary material 5 Figure S2. Haplotype network including only *T. atriceps* populations Data type occurrence Explanation note The haplotype numbers are the same as in Table 1 and Figure 8B. Every circle represents a different haplotype, the color corresponds to the geographical distribution, and the number between brackets corresponds to the number of mismatches between haplotypes. Acronyms correspond to the states in Brazil, namely: MG, Minas Gerais; PR, Paraná, RS, Rio Grande do Sul; and SC, Santa Catarina. https://binary.pensoft.net/file/534742This dataset is made available under the Open Database License (http://opendatacommons.org/licenses/odbl/1.0/). The Open Database License (ODbL) is a license agreement intended to allow users to freely share, modify, and use this Dataset while maintaining this same freedom for others, provided that the original source and author(s) are credited.Mayron E. Escárraga, John E. Lattke, Marcio R. Pie, Roberto J. Guerrero
